# Loss of Long-Term Potentiation at Hippocampal Output Synapses in Experimental Temporal Lobe Epilepsy

**DOI:** 10.3389/fnmol.2020.00143

**Published:** 2020-08-28

**Authors:** Sabine Grosser, Nadine Buck, Karl-Heinz Braunewell, Kate E. Gilling, Christian Wozny, Pawel Fidzinski, Joachim Behr

**Affiliations:** ^1^Institute of Integrative Neuroanatomy, Charité—Universitätsmedizin Berlin, Berlin, Germany; ^2^Department of Anesthesiology and Intensive Care Medicine, Charité—Universitätsmedizin Berlin, Berlin, Germany; ^3^Department of Neurophysiology, Medical Faculty, Ruhr University Bochum, Bochum, Germany; ^4^Department of Psychiatry and Psychotherapy, Charité—Universitätsmedizin Berlin, Berlin, Germany; ^5^Strathclyde Institute of Pharmacy and Biomedical Sciences, Faculty of Science, University of Strathclyde, Glasgow, United Kingdom; ^6^Department of Neurology with Experimental Neurology, Charité—Universitätsmedizin Berlin, Berlin, Germany; ^7^Department of Psychiatry and Psychotherapy, Brandenburg Medical School, Neuruppin, Germany; ^8^Department of Psychiatry and Psychotherapy, Charité—Universitätsmedizin Berlin, Berlin, Germany

**Keywords:** hippocampus, subiculum, temporal lobe epilepsy (TLE), long-term potentiation, synaptic plasticitiy

## Abstract

Patients suffering from temporal lobe epilepsy (TLE) show severe problems in hippocampus dependent memory consolidation. Memory consolidation strongly depends on an intact dialog between the hippocampus and neocortical structures. Deficits in hippocampal signal transmission are known to provoke disturbances in memory formation. In the present study, we investigate changes of synaptic plasticity at hippocampal output structures in an experimental animal model of TLE. In pilocarpine-treated rats, we found suppressed long-term potentiation (LTP) in hippocampal and parahippocampal regions such as the subiculum and the entorhinal cortex (EC). Subsequently we focused on the subiculum, serving as the major relay station between the hippocampus proper and downstream structures. In control animals, subicular pyramidal cells express different forms of LTP depending on their intrinsic firing pattern. In line with our extracellular recordings, we could show that LTP could only be induced in a minority of subicular pyramidal neurons. We demonstrate that a well-characterized cAMP-dependent signaling pathway involved in presynaptic forms of LTP is perturbed in pilocarpine-treated animals. Our findings suggest that in TLE, disturbances of synaptic plasticity may influence the information flow between the hippocampus and the neocortex.

## Introduction

It is commonly accepted that the hippocampus is involved in memory formation and consolidation, whereas permanent storage of information depends on various cortical networks (Alvarez et al., 1995; McClelland et al., 1995). An intact dialog between hippocampus and neocortex has been suggested to be critical for memory consolidation (Frankland and Bontempi, 2005) and a functional interface seems to be crucial for the translation of temporary hippocampal output into permanent memory (Squire et al., 2004). Patients with temporal lobe epilepsy (TLE) suffer from severe problems in memory consolidation and memory retrieval (Elger, 2005).

On the cellular level, activity-dependent synaptic plasticity is regarded as one of the most accepted mechanisms underlying learning and memory (Martin et al., 2000). Dependent on activation pattern of synaptic input, either a long-lasting increase or decrease in synaptic efficacy may occur (Malenka and Bear, 2004). These phenomena are referred to as long term potentiation (LTP; Bliss and Lomo, 1973) and long term depression (LTD; Dudek and Bear, 1992; Mulkey and Malenka, 1992), respectively. Anatomically, the hippocampus forms a reciprocal circuit with signals propagating from the entorhinal cortex (EC) *via* the dentate gyrus, CA3 and CA1, and the subiculum back to the EC (Groenewegen et al., 1987; Naber et al., 2001; Amaral and Lavenex, 2007). Among others, axonal projections of the CA1 mainly target the subiculum, parahippocampal regions and the EC, respectively (Cenquizca and Swanson, 2007). Particularly, the subiculum plays a major role as the principal target structure of CA1 pyramidal cells: it serves as the main relay station for outgoing hippocampal information (Burwell et al., 1995; O’Mara, 2006; Honda and Ishizuka, 2015). Previous work in rats (Deadwyler and Hampson, 2004; Menendez de la Prida et al., 2006) and humans (Gabrieli et al., 1997; Zeineh et al., 2003) has shown that the subiculum is critically involved in the encoding and retrieval of learned information.

The mechanisms that underlie the deficits in declarative memory performance in human TLE are hitherto not completely understood. To elucidate disturbances in the transfer and consolidation of information, it is of fundamental interest to understand the cellular correlates of memory formation in hippocampal output structures. Hence, using the pilocarpine-model of epilepsy, we investigated whether seizures cause alterations of LTP in these structures that may disturb the dynamic functional range of the synapses. In the present study, we focused on the expression of early LTP which is, in contrast to late LTP, independent of gene expression and protein synthesis (Frey and Morris, 1998; Kandel, 2001; Lisman et al., 2012; Herring and Nicoll, 2016).

Systemic pilocarpine administration in rats causes an acute convulsive status epilepticus and spontaneous seizures after a latent period of several weeks. The mechanisms of pilocarpine-induced ictogenesis involve cholinergic activation of excitatory neurons, depolarization blockade in certain types of inhibitory interneurons (Yi et al., 2015) and increased permeability of the blood-brain barrier (Marchi et al., 2007; Uva et al., 2008). We employed the pilocarpine-model of epilepsy, as—like in human TLE—it shows severe impairments of hippocampus-dependent acquisition and retention of spatial memory (Detour et al., 2005; Chauvière et al., 2009; Inostroza et al., 2011; Ge et al., 2016). In addition, similar to human TLE, pyramidal cells of pilocarpine-treated rats show only mild degeneration in the subiculum (Wozny et al., 2003; Knopp et al., 2005).

Using brain slices obtained from pilocarpine-treated rats, we investigated epilepsy-associated plasticity alterations at the hippocampal output with particular focus on CA1-subiculum synapses. As subicular pyramidal neurons show different forms of LTP depending on their discharge behavior (regular spiking (RS) neurons: postsynaptic LTP; bursting neurons: presynaptic LTP; Wozny et al., 2008a,b), we determined whether seizures affect synaptic plasticity at CA1-subiculum synapses in a cell type-specific manner.

Using multielectrode arrays, we found that LTP induced by high frequency stimulation was suppressed in several hippocampal output structures with the strongest effect in the subiculum. Intracellular recordings revealed that, compared to control animals, LTP was suppressed in the majority of subicular neurons affecting both, regular- and burst-spiking neurons. Our findings suggest that in epilepsy, disturbances of synaptic plasticity may influence information flow between the hippocampus and neocortical structures.

## Materials and Methods

### Pilocarpine Model of Epilepsy

The experiments were conducted in accordance with the guidelines of the European Communities Council and were approved by the Regional Berlin Animal Ethics Committee (G 0269/95, G 0328/98). Wistar-Han rats (4–6 weeks old; 130–200 g) were injected with the muscarinic agonist pilocarpine (340 mg/kg, i.p.). Peripheral cholinergic effects and lethality (9%) were reduced by pretreatment with the muscarinic acetylcholine receptor antagonist methylscopolamine (1 mg/kg, s.c.), administered 30 min prior to pilocarpine treatment. Between 10 and 30 min after pilocarpine injection, about 80% of the animals developed a generalized convulsive status epilepticus, which was terminated 90 min after seizure onset by administering diazepam (10 mg/kg, i.p.). The behavioral signs of status epilepticus disappeared approximately 15–20 min later. About 10% of the animals treated did not develop an epileptic phenotype and were excluded from experiments. Animals were kept in separate cages on a standard light/dark cycle subsequently and went through a seizure-free interval of approximately 2 weeks. Ten to 40 weeks after pilocarpine induction, rats (400–600 g) were monitored by video for three to 10 days and only animals showing spontaneous seizures were selected for experiments. Epileptic animals developed 3.3 ± 0.9 seizures per 24 h (*n* = 28 rats). Age-matched animals (400–600 g) were injected with physiological saline and used as sham controls.

### Slice Preparation and Recording Conditions

Control and pilocarpine-treated rats (age 30–55 weeks, mean 42 weeks) were decapitated under deep diethylether or 3% isofluorane anesthesia. Brains were quickly removed and washed with cooled (4°C) aerated (95% O_2_, 5% CO_2_) artificial cerebrospinal fluid (ACSF) containing in mM: NaCl 129, NaH_2_PO_4_ 1.25, NaHCO_3_ 21, KCl_3_, CaCl_2_ 1.6, MgSO_4_ 1.8, glucose 10, pH 7.4. Horizontal slices of 400 μm were obtained using a Campden vibroslicer (Loughborough, UK). Brain sections contained the ventral portions of the EC, the subiculum and the hippocampus. Slices were subsequently transferred to an interface chamber (except for network recordings) continuously perfused (~2.0 ml/min) with aerated, prewarmed (34°C) ACSF. To block GABA-A receptor-mediated response, all experiments were conducted in presence of the GABA-A receptor-antagonist bicuculline methiodide. To prevent epileptiform activity and to minimize polysynaptic activity, MgSO_4_ and CaCl_2_ concentrations of the recording solution were raised to 4 mM (Nicholls and Purves, 1970; Berry and Pentreath, 1976; Miles and Wong, 1987).

### Network Activity Recordings

Multielectrode array (MEA) recordings were performed as reported earlier (Grosser et al., 2015). In brief, slices were placed on 8 × 8 electrode grids consisting of 59 titanium nitride electrodes (60MEA200/30iR-Ti, Multichannel Systems, Reutlingen, Germany) and perfused with aerated ACSF at a rate of 5–6 ml/min. All recordings were performed under submerged conditions in a heated MEA chamber (34°C). Signals were acquired at 25 kHz sampling frequency with a MEA1060-UP-BC amplifier and digitized online with MCRack software (Multichannel Systems, Reutlingen, Germany). Only slices that displayed fEPSP response upon alvear stimulation in all regions of interest (subiculum, presubiculum, lateral and medial EC) were considered for experiments. Traces were analyzed if subicular fEPSP amplitudes of the last 5 min of recordings showed an average increase of more than 10% compared to subicular baseline amplitudes. One representative trace per region of interest showing reliable increase of fEPSP amplitudes was included. Hence, LTP was calculated by analyzing changes of fEPSP amplitudes.

### Single Cell Recordings

Intracellular recordings were performed using sharp microelectrodes (resistance 40–100 MΩ), containing 2.5 M potassium acetate. Electrodes were produced from borosilicate (Science Products GmbH, Hofheim, Germany) by a semi-automated electrode puller (Zeitz GmbH, Martinsried, Germany). Recordings were made in bridge mode using either a SEL05L or SEL10 amplifier (npi instruments, Tamm, Germany). Signals were filtered at 3 kHz and sampled at 10 kHz using an ITC18 interface and TIDA software (HEKA, Lambrecht/Pfalz, Germany). For the characterization of input resistance, hyperpolarizing current steps (200 ms, −0.1 nA) were applied. Input resistance and membrane potential were monitored during the course of each experiment and cells exhibiting more than 20% of the initial values were excluded from analysis. In general, only one cell per slice was recorded. As previously described (Wozny et al., 2008a,b), recordings were performed in the middle portion of the subiculum that receives strong synaptic input from the middle subfield of CA1 with respect to the proximo-distal axis of the CA1-subiculum region (Tamamaki and Nojyo, 1990; Amaral et al., 1991). Depolarizing current steps (0.05–2.0 nA) of 200 up to 1,000 ms duration were applied to characterize the cells’ discharge behavior (for further details see Behr et al., 1996). Subicular pyramidal cells that showed antidromic activation upon stimulation were discarded.

### Stimulation and Induction of Synaptic Plasticity

**Figure 1:**
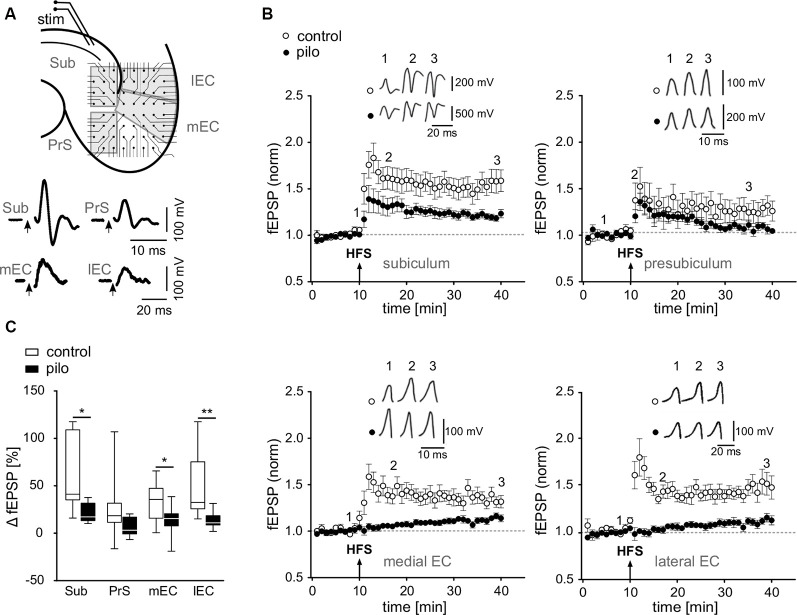
Impairment of long-term potentiation (LTP) at various hippocampal output synapses. **(A)** Top: schematic illustration of a horizontal brain slice with the subiculum and parahippocampal regions, including positioning of the stimulation electrode and multielectrode array (MEA) recording electrodes [Sub, subiculum; PrS, presubiculum; lEC, lateral entorhinal cortex (EC); mEC, medial entorhinal cortex]. Bottom: corresponding sample traces of fEPSPs recorded in slices of control animals. **(B)** fEPSP time courses obtained from MEA recordings in slices from control and pilocarpine-treated animals upon alvear stimulation in CA1 as illustrated in **(A)** Arrows show time point of HFS. **(C)** Summary of the relative fEPSP change upon HFS as presented in panel **(B)** shown as box plots with whiskers from minimum to maximum and line at median for all recordings (Control: Sub: 56.9 ± 11.1%; PrS: 25 ± 9.4%; mEC: 32.6 ± 6.1%; lEC: 50.8 ± 10.9%; *n* = 11; Pilo: Sub: 20.6 ± 3.1%; PrS: 6.6 ± 3%; mEC: 13.7 ± 4.6%; lEC: 13.3 ± 2.8%; *n* = 10). Statistical significance was set to *p < 0.05 and **p < 0.01.

EPSPs and fEPSPs were evoked upon alvear stimulation in the distal CA1 region, close to proximal subiculum (Figure 1A). Responses were evoked using 100 μs pulses every 20 s (EPSP recordings) or every 10 s (fEPSPs recordings) with glass-insulated bipolar platinum wire electrodes (tip separation 50–100 μm). The stimulus intensity was set to 40–60% of the maximum response (intracellular: largest EPSP amplitude that did not evoke an action potential; extracellular: maximum population spike amplitude). Input-output curves were generated by plotting normalized peak amplitudes vs. the stimulation intensity.

To induce LTP, four high-frequency tetani were applied at 100 Hz for 1 s with 10 s inter-train intervals. The magnitude of LTP was determined by calculating the ratio of EPSP or fEPSP amplitude, respectively, collected during the last 5 min of each experiment and the corresponding average baseline amplitude. Paired-pulse facilitation (PPF) was investigated by analyzing the ratio of the second to the first synaptic response (EPSP2/EPSP1; inter-stimulus interval: 50 ms) 5 min before and 25–30 min after the conditioning stimulus.

### Drugs

The following drugs were bath applied: bicuculline, 10 μM (purchased from Sigma–Aldrich, Taufkirchen, Germany) and forskolin, 50 μM (purchased from Tocris/Biotrend, Cologne, Germany).

### SDS-PAGE and Western Blot Analysis

Tissue samples of control and pilocarpine-treated animals containing only the subiculum were homogenized at 4°C in extraction buffer [150 mM NaCl, 50 mM Tris, 10 mM HEPES, 1% Triton X-100, 1% EGEPAL, proteinase inhibitor cocktail (Roche)]. Homogenates were centrifuged at 20,000 *g* for 15 min and the resulting supernatant was used for SDS-PAGE and protein determinations with the BCA assay (Pierce, Rockford, IL, USA). Western blot analysis was performed using 5–20% gradient SDS-PAGEs. Gels were electro blotted on PVDF membranes (Roth, Karlsruhe, Germany) for 2 h and membranes subsequently blocked for 1 h in TBST buffer (100 mM Tris-HCl; 0.9% NaCl, 1% Tween 20, pH 7.4) containing 5% non-fat dry milk. Blots were incubated overnight at 4°C with specific primary antibodies (anti AC-1 1:200, SC-25743, Santa Cruz Biotechnology, Santa Cruz, CA, USA, anti-Actin 1:5,000, Santa Cruz Biotechnology, Santa Cruz, CA, USA) and immunoreactivity was visualized using HRP-coupled goat anti-rabbit or goat anti-mouse secondary antibodies (Santa Cruz Biotechnology, Santa Cruz, CA, USA). Quantification of Western blots was done by densitometric analysis using the NIH Image program v1.61. The relative expression levels of adenylyl cyclase 1 (AC1) and actin as well as the ratio of AC1 and actin are expressed as mean values ± standard error of mean (SEM).

### Statistics

Data were expressed as means ± SEM Statistical evaluation was performed by applying Shapiro-Wilk normality test to assess normal distribution and then by using student’s *t*-test, Mann–Whitney test or 2-way ANOVA where appropriate and not stated otherwise (GraphPad Prism, La Jolla, CA, USA). Significance level was set to *p* < 0.05. For relative changes of fEPSP and EPSP amplitudes, data was normalized and the last 5 min of each recording was compared to 5 min of stable baseline recording. Data is generally represented as box plots with whiskery extending from minimum to maximum and line at median, if not stated otherwise.

## Results

### Suppression of LTP at Hippocampal Output Synapses

To investigate plasticity at hippocampal output synapses in an animal model of epilepsy, we focused on regions of interest that receive inputs from the hippocampus proper, including the subiculum, the presubiculum, and the medial and lateral EC. Using multielectrode arrays, we simultaneously recorded fEPSPs from several hippocampal regions upon stimulation of alvear CA1 fibers (Figure 1A). Stable fEPSPs upon alvear stimulation could be evoked in slices from both, control and pilocarpine-treated animals in the subiculum, the presubiculum and in the deep layers of both, the medial and the lateral EC. In control slices, tetanic stimulation of alvear fibers evoked robust LTP in all investigated regions with the strongest effect in the subiculum (Sub: 56.9 ± 11.1%; PrS: 25 ± 9.4%; mEC: 32.6 ± 6.1%; lEC: 50.8 ± 10.9%; *n* = 11; Figures 1B,C). In slices from pilocarpine-treated rats, however, LTP was strongly suppressed in the subiculum, the presubiculum and both, the medial and lateral EC (Sub: 20.6 ± 3.1%; PrS: 6.6 ± 3%; mEC: 13.7 ± 4.6%; lEC: 13.3 ± 2.8%; *n* = 10; Figures 1B,C). Our results demonstrate a significant, epilepsy-induced loss of LTP at the hippocampal output (*F* = 32.1, *p* < 0.001) as well as significant region-specific differences (*F* = 3.6, *p* < 0.018), though the interaction between these sets of data was not significant (2-way ANOVA). A Bonferroni *post hoc* test revealed significant pairwise differences between control and pilocarpine-treated cells in the subiculum, the medial and lateral EC (Sub: −31.2%, *p* < 0.05; PrS: −20.7%, *p* = n.s.; mEC: −26.4%, *p* < 0.05; lEC: −38.3%, *p* < 0.01).

### Membrane and Synaptic Properties of Subicular Pyramidal Cells

**Figure 2:**
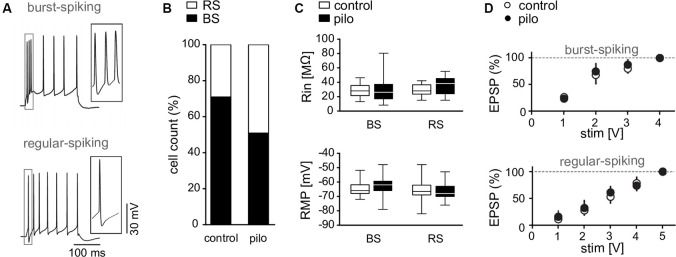
Membrane properties of subicular pyramidal cells. **(A)** Discharge behavior of RS and BS pyramidal cells upon depolarizing current pulses (300 pA). **(B)** Cell count of burst spiking (BS) and regular spiking (RS) fractions in control and pilocarpine-treated animals showing a decreased fraction of BS cells in pilocarpine-treated animals (Control BS: 71%, Control RS: 29%, *n* = 69; BS Pilo: 50.9%, RS Pilo: 49.1%, *n* = 75; *p* = 0.01, Fischer’s exact test). **(C)** Input resistance (Control BS: Rin = 28.5 ± 2.1 MΩ, *n* = 19, Pilo BS: Rin = 29.1 ± 3.1 MΩ, *n* = 29, *p* = n.s.; Control RS: Rin = 30 ± 1.8 MΩ, *n* = 17, Pilo RS: Rin = 36.2 ± 2.9 MΩ, *n* = 23, *p* = n.s.) and resting membrane potential (Control BS: RMP = −64.4 ± 1.2 mV, *n* = 19, Pilo BS: RMP = −62.6 ± 1.2 mV, *n* = 29, *p* = n.s.; Control RS: RMP = −66.4 ± 2 mV, *n* = 17, Pilo RS: RMP = −65.7 ± 1.4 mV, *n* = 23) of subicular neurons are not different between slices from control and pilocarpine-treated rats. Here shown as box plots with whiskers from minimum to maximum and line at median. **(D)** Input-output curves of BS and RS cells obtained from control and pilocarpine-treated animals (Control: BS: *n* = 23, RS: *n* = 5; Pilo: BS: *n* = 7; RS: *n* = 7).

For a more detailed characterization of LTP suppression in epileptic animals, we performed single cell recordings in the subiculum, which is considered as the main target of CA1 pyramidal cells and displayed a robust LTP in MEA recordings. As reported earlier (Amaral et al., 1991; Mattia et al., 1993; Harris and Stewart, 2001a; Knopp et al., 2005; Chauvière et al., 2009) subicular pyramidal cells can be divided in two distinct classes according to their gene expression, long-range projections and electrophysiological properties. In accordance with previous reports (Mattia et al., 1993; Stewart and Wong, 1993; Taube, 1993; Behr et al., 1996; Staff et al., 2000; O’Mara et al., 2001; Wellmer et al., 2002; Menendez de la Prida et al., 2003), subicular pyramidal cells responded to depolarizing pulses either by burst spiking (BS) behavior with bursts of 2–3 action potentials followed by single action potentials or regular spiking (RS) behavior with trains of single action potentials (Figure 2A). In control preparations, BS cells outnumbered RS cells by approximately two to one. In contrast, in slices from pilocarpine-treated animals, the proportion of BS to RS cells was markedly changed (Control: 71% BS cells, 29% RS cells, *n* = 69; Pilocarpine: 50.9% BS cells, 41.1% RS cells, *n* = 112; *p* < 0.01, Fisher’s exact test, Figure 2B), suggesting that epileptogenesis either induced a neuronal loss preferentially in the BS cell population or a switch from BS cell to RS cell behavior. Consequently, in epileptic tissue, subicular pyramidal cells with regular-spiking behavior were regarded as putative RS cells and cells with burst-spiking behavior as putative BS cells.

Neither input resistance (Control BS: Rin = 28.5 ± 2.1 MΩ, *n* = 19, Control RS: Rin = 30 ± 1.8 MΩ, *n* = 17; Pilocarpine BS: Rin = 29.1 ± 3.1 MΩ, *n* = 29, Pilocarpine RS: Rin = 36.2 ± 2.9 MΩ, *n* = 23, Figure 2C) nor resting membrane potential (Control BS: RMP = −64.4 ± 1.2 mV, *n* = 19, Control RS: RMP = −66.4 ± 2 mV, *n* = 17, Pilocarpine BS: RMP = −62.6 ± 1.2 mV, *n* = 29, Pilocarpine RS: RMP = −65.7 ± 1.4 mV, *n* = 23; Figure 2C) were different between control and epileptic tissue (*F* = 0.8; *p* = n.s.) or showed cell type specific characteristics (*F* = 3.3, *p* = n.s., Bonferroni *post hoc* test, 2-way ANOVA). Also, EPSP kinetics determined by rise and decay time constants and the transmembrane charge movement were not different between cell types or animal groups (not shown). As subicular tissue from pilocarpine-treated animals displays increased excitability (Grosser et al., 2015), we investigated EPSPs in both cell types at various stimulation intensities. We found that the input-output behavior for subthreshold EPSPs in single cell recordings was not changed in slices from pilocarpine-treated rats as compared to control preparations (Figure 2D), indicating that increased excitability in the subiculum from epileptic animals is rather due to network alterations than to an increased synaptic excitability at CA1-subiculum synapses. However, axonal sprouting seems not mandatory for the development of the enhanced network excitability. Rather, pilocarpine-induced seizures cause an unmasking and/or strengthening of synaptic connectivity within the recurrent subicular network (Knopp et al., 2008).

### Cell-Specific Loss of Activity-Dependent LTP in the Subiculum

Next, we investigated whether LTP suppression observed in fEPSP/MEA recordings in the subiculum from pilocarpine-treated rats was reproducible at the single cell level. RS and BS cells show fundamentally different forms of LTP at CA1-subiculum synapses with postsynaptic NMDA receptor dependent LTP in RS cells and presynaptic LTP in BS cells with involvement of the presynaptic cAMP-cascade (Wozny et al., 2008a,b).

**Figure 3:**
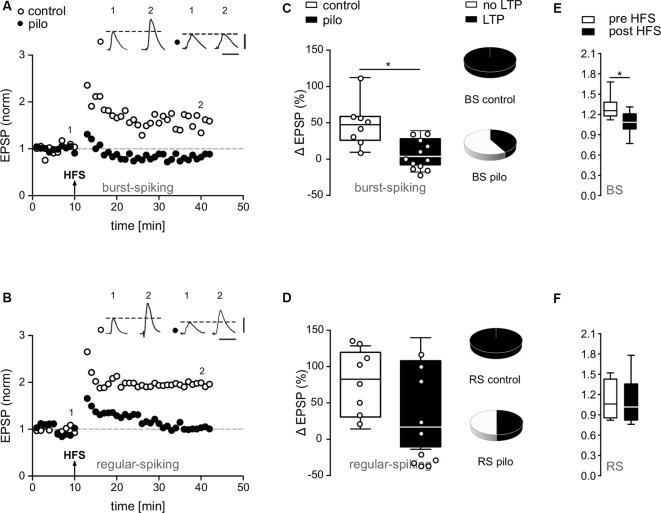
Impairment of synaptic plasticity in subicular pyramidal cells of pilocarpine-treated animals. **(A,B)** EPSP time courses from single cell recordings in subicular BS and RS cells before and after HFS. Note the loss of LTP in putative subicular BS and RS cells of pilocarpine-treated animals after HFS. **(C,D)** Summary of relative changes in synaptic strength upon HFS shown as box plots with whiskers from minimum to maximum and line at median (Control BS: 44.8 ± 1.4%, *n* = 8, Pilo BS: 7.6 ± 2.1%, *n* = 12, *p* = 0.004; Control RS: 75.9 ± 15.7%, *n* = 8, Pilo RS: 40.4 ± 19%, *n* = 10, *p* = n.s.). Pie charts depict fractions of BS and RS cells in control and pilocarpine-treated animals showing LTP. While in control animals all BS and RS cells showed a significant degree of LTP, in pilocarpine-treated animals, only five out of 10 putative RS cells and five out of 12 putative BS cells showed LTP. **(E,F)** The expression of LTP in control animals was accompanied by a change in PPF in BS but not in RS cells (Control BS: Pre HFS PPR = 1.3 ± 0.06, *n* = 8; Post HFS PPR = 1.1 ± 0.06, *n* = 8, *p* = 0.03, *t*-test; Control RS: Pre HFS PPR = 1.1 ± 0.1, *n* = 8; Post HFS PPR = 1.1 ± 0.1, *n* = 8, *p* = n.s., *t*-test), suggesting a presynaptic mechanism. Data is shown as box plots with whiskers from minimum to maximum and line at median. Statistical significance was set to *p < 0.05.

In the present study, high frequency stimulation of alvear fibers in the CA1 caused stable LTP in BS and RS cells in control animals (Control BS: 48.5 ± 10.9%, *n* = 8, Control RS: 75.9 ± 15.7%, *n* = 8; Figures 3A–D). In contrast to control animals, in pilocarpine-treated animals about half of subicular cells failed to express LTP (Figures 3A–D). While RS cells from pilocarpine-treated animals still expressed a significantly reduced but stable LTP compared to control animals (*p* = n.s., *t*-test), LTP in BS cells was completely suppressed in pilocarpine-treated animals (*p* < 0.05, Mann–Whitney test, Pilo BS: 8.9 ± 5.7%, *n* = 12; Pilo RS: 40.4 ± 19%, *n* = 10; Figures 3A–D). The failure to express LTP was not dependent on the intrinsic firing pattern as both putative BS and putative RS cells did not display LTP in approximately 50–60% of our recordings (Figures 3C,D). Of note, this reduction is in line with the diminished LTP observed in the extracellular recordings.

The PPF provides an indirect measure of the ability of synapses to alter transmitter release between two closely spaced stimuli and depends on residual calcium levels in the presynaptic terminal (Zucker and Regehr, 2002). As reported earlier in younger animals (Wozny et al., 2008b), LTP in BS but not in RS cells was accompanied by a substantial change in PPF (Figures 3E,F), indicating a presynaptic expression (Control BS: Pre HFS PPR = 1.3 ± 0.1, *n* = 8; Post HFS PPR = 1.1 ± 0.1, *n* = 8, *p* = 0.03, *t*-test; Control RS: Pre HFS PPR = 1.1 ± 0.1, *n* = 8; Post HFS PPR = 1.1 ± 0.1, *n* = 8, *p* = n.s., *t*-test).

### Short-Term Plasticity in BS Cells

**Figure 4:**
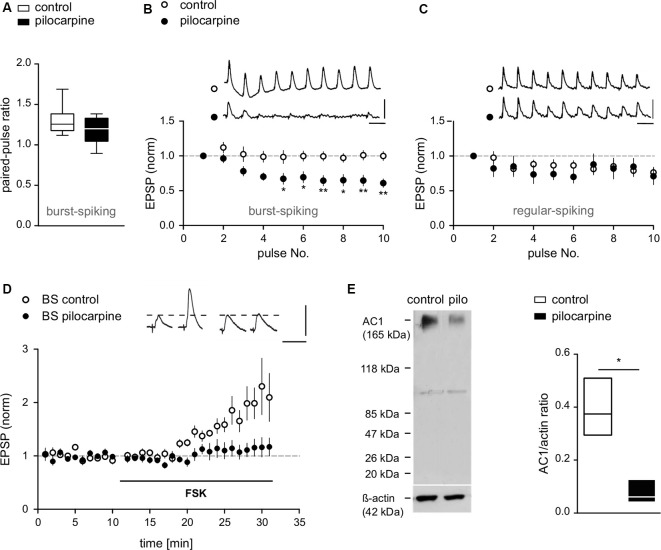
Compromised presynaptic release machinery in BS cells from pilocarpine-treated animals. **(A)** PPF of EPSPs recorded from BS cells in control and pilocarpine-treated animals shown as box plots with whiskers from minimum to maximum and line at median (Control BS: PPR = 1.3 ± 0.06, *n* = 8, Pilo BS: PPR = 1.2 ± 0.04, *n* = 12, *p* = n.s). **(B)** EPSP responses upon repetitive stimulation (10 pulses at 10 Hz) in BS cells from control and pilocarpine-treated rats. In pilocarpine-treated animals, we observed a strong decline of the EPSP amplitude upon the 4th to 10th stimulus (Control BS: −0.1 ± 1%, *n* = 8, Pilo BS: −36.5 ± 1.3%, *n* = 10). **(C)** EPSP responses upon repetitive stimulation (10 pulses at 10 Hz) in RS cells from control and pilocarpine-treated rats. In control animals (*n* = 8), six cells showed weak depression, one cell showed no effect and one cell showed an increase in EPSP amplitude. In pilocarpine-treated animals (*n* = 9), seven cells showed weak depression, one cell showed no effect and one cell showed an increase in EPSP amplitude. There was no statistical difference between control and pilocarpine-treated animals (Control RS: −19.8 ± 0.9%, *n* = 8, Pilo RS: −20.7 ± 4.2%, *n* = 9). Scale bars: 5 mV and 100 ms. **(D)** In control BS cells, application of forskolin (FSK) caused a potentiation of EPSPs (FSK: 100.4 ± 7.9%, *n* = 7) while in BS cells from pilocarpine-treated animals, forskolin failed to induce LTP (FSK: 13.4 ± 1.3%, *n* = 8). Scale bars: 5 mV and 50 ms. **(E)** Left: protein immunoblot analysis of AC1 expression in subicular slices. Protein expression of AC1 was detected in membrane-enriched protein extracts from the subiculum of control and pilocarpine-treated rats. Equal loading conditions were verified by immunodetection for actin. An immunoreactive band at approximately 123 kDa, representing AC1 protein was detected with reduced expression in pilocarpine-treated samples. Sizes of relevant marker proteins in kDa are shown in the left margin. Faint lower bands at approximately 100 kDa are of unknown origin, possibly due to protein degradation. Right: quantification of AC1 and actin immunreactive bands in membrane-enriched protein extracts from the subiculum of control and pilocarpine-treated rats shown as floating bars with line at median (AC1/actin ratio Control: 0.39 ± 0.06, *n* = 3; AC1/actin ratio Pilo: 0.08 ± 0.03, *n* = 3, *p* = 0.04). Statistical significance was set to *p < 0.05, **p < 0.01.

Given the presynaptic induction mechanism of LTP in BS cells in control animals (Wozny et al., 2008a,b), the loss of LTP in some cells points towards a disturbance of the presynaptic signaling cascade. To investigate the presynaptic release machinery, we first studied PPF in BS cells obtained from control and pilocarpine-treated animals, respectively, by applying two pulses with 50 ms inter-stimulus intervals. We found no significant difference between these animal groups (Control BS: PPR = 1.3 ± 0.06, *n* = 8, Pilocarpine BS: PPR = 1.2 ± 0.04, *n* = 12, *p* = n.s., *t*-test; Figure 4A). As repetitive stimulation is more sensitive than PPF to detect deficits in presynaptic release, we next used a high-frequency stimulation protocol (10 pulses at 10 Hz, 100 ms inter-stimulus interval) below the threshold for the induction of LTP to investigate differences in the release machinery in more detail. For BS cells, we observed a significant decline of the EPSP amplitude upon the 4th to 10th stimulus in pilocarpine-treated but not in control animals (Control BS: −0.1 ± 1%, *n* = 8, Pilocarpine BS: −36.5 ± 1.3%, *n* = 10, *F* = 72.1, *p* < 0.001, 2-way ANOVA; Figure 4B), suggesting that the pilocarpine treatment and the consecutive epileptogenesis has negatively affected the release machinery. For RS and putative RS cells, we found no significant differences in EPSP amplitudes upon repetitive stimulation between control and pilocarpine-treated animals (Control RS: −19.8 ± 0.9%, *n* = 8, Pilocarpine RS: −20.7 ± 4.2%, *n* = 9, *F* = 1.3, *p* = n.s., 2-way ANOVA; Figure 4C).

### Loss of Forskolin-Induced LTP in BS Cells

In previous work, we showed that activation of adenylyl cyclase (AC) by forskolin (FSK) or application of the cAMP-analogue Sp-5, 6-DCl-cBIMPS mimics and occludes presynaptic LTP in subicular BS neurons. This effect can be inhibited by various inhibitors of the cAMP-protein kinase A (PKA) cascade (Wozny et al., 2008b). Here, FSK consistently enhanced EPSPs in control BS cells (FSK: 100.4 ± 7.9%, *n* = 7; Figure 4D). Consistent with the loss of tetanus-induced LTP in epileptic tissue, forskolin likewise failed to induce LTP in putative BS cells from pilocarpine-treated animals (FSK: 13.4 ± 1.3%, *n* = 8; Figure 4D), showing that pharmacological activation of AC does not increase transmitter release in these animals.

### AC1 Expression in Control and Pilocarpine-Treated Animals

Several forms of synaptic plasticity depend on AC-cAMP signaling. Two of the eight known forms of AC are activated by calcium, AC1 and AC8 (for review see Ferguson and Storm, 2004). As AC1 is the most abundant isoform in the hippocampal formation and shows higher sensitivity towards calcium than AC8 (Wang and Storm, 2003), here, we focused on AC1.

Western blot analysis of subicular tissue revealed AC1 protein expression in control animals, but a strong reduction of AC1 in slices of pilocarpine-treated animals (Figure 4E). The control protein actin was not obviously affected by pilocarpine treatment. Quantification of AC1 protein levels normalized to control protein expression (lower panel) showed significant differences, indicating that the observed reduction in the AC1 expression was specific, and was not caused by a general down-regulation of protein expression in pilocarpine-treated animals (AC1/actin ratio Control: 0.39 ± 0.06, *n* = 3; AC1/actin ratio Pilocarpine: 0.08 ± 0.03, *n* = 3, *p* = 0.04; Figure 4E).

## Discussion

In the present study performed in brain slices from pilocarpine-treated rats, we found a severe impairment of synaptic plasticity at CA1 synapses targeting various output regions such as the subiculum, the presubiculum as well as the medial and the lateral EC. At CA1-subiculum synapses, single cell recordings further revealed an impairment of synaptic plasticity in both subicular cell types. In addition, we observed a reduced BS/RS cell ratio, though under epileptic conditions bursting is often rather increased mainly due to postsynaptic mechanisms (Sanabria et al., 2001; Wellmer et al., 2002). Taking into account that a switch from BS to RS behavior might occur during epileptogenesis, in the present study, subicular pyramidal cells from epileptic tissue were regarded as putative BS or RS cells, respectively.

Studies on resting properties of subicular pyramidal neurons could not find robust and consistent differences between RS and BS cells (Behr et al., 1996; Staff et al., 2000; Menendez de la Prida et al., 2003). While in our previous study (Knopp et al., 2005) patch-clamp recordings showed differences in basic membrane properties between control and pilocarpine-treated animals, here no changes in basic membrane properties of RS or BS cells were detected. Knowledge of possible alterations in basic membrane properties is important for the interpretation of changes in excitability. In the present work, we favored sharp microelectrode recordings as they better preserve intracellular cascades and thus facilitate reliable induction of synaptic plasticity in adult hippocampal tissue. This type of recording, however, carries the limitation of leak currents that might decrease the input resistance (Staley et al., 1992). Therefore, in the present study, interpretation of potential differences in input resistance between control and pilocarpine-treated animals was limited and might have been masked for methodological reasons. The input resistances are in line with previously published data showing rather low input resistances. Menendez de la Prida et al. (2003) reported an input resistance of 82 ± 31 MΩ for bursting neurons, whereas Staff et al. (2000) reported an even lower input resistance of 36 ± 3 MΩ for bursting neurons using whole-cell recordings.

The disruption of presynaptic LTP including unchanged PPF and robust EPSP depression during repetitive stimulation point to an impairment of the presynaptic release machinery at CA1-BS cell synapses of pilocarpine-treated animals. In general, these synapses are able to increase their release probability when stimulated at higher frequencies. This notion is further supported by experimental elevation of cAMP by forskolin, which caused a strong EPSP increase in BS cells of control, but not pilocarpine-treated animals. Finally, protein expression of AC1 was strongly reduced in subicular slices from pilocarpine-treated animals indicating that a decrease in the expression of AC1 might be causative for cAMP-dependent LTP occlusion in BS cells. The observed EPSP depression upon repetitive stimulation might have different reasons such as low vesicle pool size, impairment of vesicle-recycling mechanisms, or reduced calcium entry. As the input-output curves of BS cells showed no difference between control and pilocarpine-treated animals, initial transmitter release probability and number of synaptic terminals seem to be unaffected.

Our results indicate a high sensitivity of the cAMP-PKA-dependent LTP in BS cells against epileptogenesis and seizures. These findings are in concert with previous work demonstrating that kainate-induced seizures impair presynaptic function of the cAMP-PKA signaling cascade at mossy fiber-CA3 synapses (Goussakov et al., 2000). Genetic disruption studies indicate that AC1 activity is required for presynaptic, tetanically induced LTP (Villacres et al., 1998; Wong et al., 1999). However, in knockout animals of AC1 and AC8 chemical induction by forskolin is still capable of inducing postsynaptic LTP at CA1 synapses (Wong et al., 1999). These contrary findings raise the question whether reduction in AC1 expression or activity underlie the loss of presynaptic LTP in the epileptic hippocampus. In agreement with localization studies showing a widely distributed hippocampal AC1 expression (Conti et al., 2007), Western blot analysis revealed abundant AC1 expression in control hippocampal slices, which however was reduced in slices from pilocarpine-treated animals tissue. Reduced AC1 content without concomitant protein loss as demonstrated by comparable actin levels could be either caused by reduced AC1 content per synapse or a reduced synapse count in epileptic animals, potentially secondary to CA1 cell loss. As both mechanisms would result in an LTP impairment, experimental verification using synaptic markers (Wang et al., 2002) will be required to prove which mechanism might be involved here. However, as the input-output behavior was not changed in pilocarpine-treated animals as compared to controls, a reduced level of AC1 due to a loss of synapses seems to be unlikely.

Importantly, AC1 is not only the most abundant isoform in the hippocampal formation, it also shows higher sensitivity towards calcium than, for example, AC8 (Wang and Storm, 2003). Loss of AC1 expression together with loss of cAMP-dependent LTP in pilocarpine-treated rats indicate that these aspects might be related. To prove this point, however, further studies in genetically modified (AC1 disruption) animals treated with pilocarpine would be required. The exact mechanisms underlying reduced expression of AC1 in epileptic animals are not known. Downregulation of AC1 mRNA has been demonstrated in response to synaptic deafferentation and reinnervation, a common phenomenon in epilepsy (Laurent-Demir et al., 2000). Alternatively, as AC1 has been implicated in NMDA receptor-dependent excitotoxicity (Wang et al., 2007), a downregulation as part of a neuroprotective feedback regulation during epilepsy might be regarded. Interestingly, deficiencies in hippocampus-dependent learning (Wu et al., 1995; Wong et al., 1999) occur in both, TLE patients and in AC1 knock-out and accordingly AC1/AC8-double knock-out mice (Wu et al., 1995; Wong et al., 1999). We therefore speculate that AC1 downregulation might be a factor contributing to the development of declarative memory impairments in patients.

Like in BS cells, LTP in RS cells was blocked in nearly half of the recorded cells obtained from pilocarpine-treated animals. Given that LTP in RS cells is expressed postsynaptically (Fidzinski et al., 2008; Wozny et al., 2008b), our findings support the notion that in addition to the discussed alterations of the presynaptic release machinery, epileptogenesis seems to impair postsynaptic forms of synaptic plasticity as well. Future studies will have to address this phenomenon and the underlying postsynaptic mechanisms in more detail.

Considering subicular function, *in vivo* experiments indicate that the subiculum reacts to new information earlier than the hippocampus proper, which in rodents encompasses a time frame of 10–15 s (Deadwyler and Hampson, 2004). Subicular activation is then succeeded by an increasing participation of the CA1 network. These data support the hypothesis that the subiculum acts as a detector and distributor of sensory information that takes into account the novelty and relevance of signals arriving from the CA1 (Burwell, 2006; Naber et al., 2006). Subicular pyramidal cells relay CA1 output to a variety of cortical and subcortical structures (Witter, 2006). BS and RS cells are spatially distributed in the proximo-to-distal and deep-to-superficial axes of the subiculum (Greene and Totterdell, 1997; Staff et al., 2000; Harris and Stewart, 2001a; Menendez de la Prida et al., 2003) and show specific and bidirectional EC-subiculum connectivity (Harris and Stewart, 2001b) with RS cells connected to the lateral EC and BS cells connected mainly to the medial EC (Naber and Witter, 1998; Ishizuka, 2001). In addition to this strong EC connection, RS and BS cells projections also differ at more distant locations (i.e., BS cells projecting to the retrosplenial cortex and BS cells projecting to the Ncl. accumbens), indicating that BS and RS cells may process different and complementary functions such as memory or emotion. Disturbance of synaptic plasticity in both cell types in epileptic animals therefore is expected to impair this differential processing of information from the hippocampus to subsequent cortical and subcortical brain regions and might contribute to the neuropsychological comorbidities observed in patients suffering from TLE. As the present study focused on early protein synthesis-independent LTP, no conclusion about long lasting effects can be drawn. Though there is compelling evidence for the role of early LTP in learning and memory (Nicoll, 2017), future studies will have to add further information on the effect of seizure-activity on long LTP and hippocampal function.

## Data Availability Statement

The raw data supporting the conclusions of this article will be made available by the authors, without undue reservation.

## Ethics Statement

The animal study was reviewed and approved by Berlin Animal Ethics Committee (G 0269/95, G 0328/98).

## Author Contributions

SG carried out MEA experiments. NB, K-HB, KG, and PF carried out patch-clamp experiments. SG, PF, JB, and CW contributed to the interpretation of the results. PF, SG, and JB took the lead in writing the manuscript. JB and CW conceived the study and were in charge of overall direction and planning. All authors provided critical feedback and helped shape the research, analysis and manuscript.

## Conflict of Interest

The authors declare that the research was conducted in the absence of any commercial or financial relationships that could be construed as a potential conflict of interest.

## References

[B1] Alvarez P. Zola-Morgan S. Squire L. R. (1995). Damage limited to the hippocampal region produces long-lasting memory impairment in monkeys. J. Neurosci. 15, 3796–3807. 10.1523/jneurosci.15-05-03796.1995 7751947 PMC6578197

[B2] Amaral D. Lavenex P. (2007). “Hippocampal neuroanatomy,” in The Hippocampus Book, eds Andersen P. Morris R. Amaral D. Bliss T. O’Keefe J. (New York, NY: Oxford University Press), 37–114.

[B3] Amaral D. G. Dolorfo C. Alvarez-Royo P. (1991). Organization of CA1 projections to the subiculum: a PHA-L analysis in the rat. Hippocampus 1, 415–435. 10.1002/hipo.450010410 1727001

[B4] Behr J. Empson R. M. Schmitz D. Gloveli T. Heinemann U. (1996). Electrophysiological properties of rat subicular neurons *in vitro*. Neurosci. Lett. 220, 41–44. 10.1016/s0304-3940(96)13242-0 8977144

[B5] Berry M. S. Pentreath V. W. (1976). Criteria for distinguishing between monosynaptic and polysynaptic transmission. Brain Res. 105, 1–20. 10.1016/0006-8993(76)90919-7 175886

[B6] Bliss T. Lomo T. (1973). Long-lasting potentiation of synaptic transmission in the dentate area of the anaesthetized rabbit following stimulation of the perforant path. J. Physiol. 232, 331–356. 10.1113/jphysiol.1973.sp010273 4727084 PMC1350458

[B7] Burwell R. D. Witter M. P. Amaral D. G. (1995). Perirhinal and postrhinal cortices of the rat: a review of the neuroanatomical literature and comparison with findings from the monkey brain. Hippocampus 5, 390–408. 10.1002/hipo.450050503 8773253

[B8] Burwell R. D. (2006). The parahippocampal region: corticocortical connectivity. Annu. N Y Acad. Sci. 911, 25–42. 10.1111/j.1749-6632.2000.tb06717.x 10911865

[B9] Cenquizca L. A. Swanson L. W. (2007). Spatial organization of direct hippocampal field CA1 axonal projections to the rest of the cerebral cortex. Brain Res. Rev. 56, 1–26. 10.1016/j.brainresrev.2007.05.002 17559940 PMC2171036

[B10] Chauvière L. Rafrafi N. Thinus-Blanc C. Bartolomei F. Esclapez M. Bernard C. (2009). Early deficits in spatial memory and theta rhythm in experimental temporal lobe epilepsy. J. Neurosci. 29, 5402–5410. 10.1523/JNEUROSCI.4699-08.2009 19403808 PMC6665868

[B11] Conti A. C. Maas J. W. Jr. Muglia L. M. Dave B. A. Vogt S. K. Tran T. T.. (2007). Distinct regional and subcellular localization of adenylyl cyclases type 1 and 8 in mouse brain. Neuroscience 146, 713–729. 10.1016/j.neuroscience.2007.01.045 17335981 PMC1939925

[B12] Deadwyler S. A. Hampson R. E. (2004). Differential but complementary mnemonic functions of the hippocampus and subiculum. Neuron 42, 465–476. 10.1016/s0896-6273(04)00195-3 15134642

[B13] Detour J. Schroeder H. Desor D. Nehlig A. (2005). A 5-month period of epilepsy impairs spatial memory, decreases anxiety, but spares object recognition in the lithium-pilocarpine model in adult rats. Epilepsia 46, 499–508. 10.1111/j.0013-9580.2005.38704.x 15816943

[B14] Dudek S. M. Bear M. F. (1992). Homosynaptic long-term depression in area CA1 of hippocampus and effects of N-methyl-D-aspartate receptor blockade. Proc. Nat. Acad. Sci. U S A. 89, 4363–4367. 10.1073/pnas.89.10.4363 1350090 PMC49082

[B15] Elger C. E. (2005). Epilepsy: a model for the study of brain function. Lancet Neurol. 4:3. 10.1016/S1474-4422(04)00943-3 15620845

[B300] Ferguson G. D. Storm D. R. (2004). Why calcium-stimulated adenylyl cyclases? Physiology (Bethesda) 19, 271–276. 10.1152/physiol.00010.2004 15381755

[B16] Fidzinski P. Shor O. Behr J. (2008). Target-cell-specific bidirectional synaptic plasticity at hippocampal output synapses. Eur. J. Neurosci. 27, 1111–1118. 10.1111/j.1460-9568.2008.06089.x 18312585

[B17] Frankland P. W. Bontempi B. (2005). The organization of recent and remote memories. Nat. Rev. Neurosci. 6, 119–130. 10.1038/nrn1607 15685217

[B18] Frey U. Morris R. G. M. (1998). Synaptic tagging: implications for late maintenance of hippocampal long-term potentiation. Trends Neurosci. 21, 181–188. 10.1016/s0166-2236(97)01189-2 9610879

[B19] Gabrieli J. D. Brewer J. B. Desmond J. E. Glover G. H. (1997). Separate neural bases of two fundamental memory processes in the human medial temporal lobe. Science 276, 264–266. 10.1126/science.276.5310.264 9092477

[B20] Ge Y.-X. Tian X.-Z. Lin Y.-Y. Liu X.-Y. (2016). Chronic treatment with levetiracetam reverses deficits in hippocampal LTP *in vivo* in experimental temporal lobe epilepsy rats. Neurosci. Lett. 628, 194–200. 10.1016/j.neulet.2016.06.043 27345386

[B21] Goussakov I. V. Fink K. Elger C. E. Beck H. (2000). Metaplasticity of mossy fiber synaptic transmission involves altered release probability. J. Neurosci. 20, 3434–3441. 10.1523/jneurosci.20-09-03434.2000 10777806 PMC6773116

[B22] Greene J. Totterdell S. (1997). Morphology and distribution of electrophysiologically defined classes of pyramidal and nonpyramidal neurons in rat ventral subiculum *in vitro*. J. Comp. Neurol. 380, 395–408. 10.1002/(sici)1096-9861(19970414)380:3<395::aid-cne8>3.0.co;2-y 9087521

[B23] Groenewegen H. J. Vermeulen-Van der Zee E. te Kortschot A. Witter M. P. (1987). Organization of the projections from the subiculum to the ventral striatum in the rat. A study using anterograde transport of phaseolus vulgaris leucoagglutinin. Neuroscience 23, 103–120. 10.1016/0306-4522(87)90275-2 3683859

[B24] Grosser S. Hollnagel J. O. Gilling K. E. Bartsch J. C. Heinemann U. Behr J. (2015). Gating of hippocampal output by β-adrenergic receptor activation in the pilocarpine model of epilepsy. Neuroscience 286, 325–337. 10.1016/j.neuroscience.2014.11.055 25498224

[B25] Harris E. Stewart M. (2001a). Intrinsic connectivity of the rat subiculum: II. Properties of synchronous spontaneous activity and a demonstration of multiple generator regions. J. Comp. Neurol. 435, 506–518. 10.1002/cne.1047 11406829 PMC1592136

[B26] Harris E. Stewart M. (2001b). Propagation of synchronous epileptiform events from subiculum backward into area CA1 of rat brain slices. Brain Res. 895, 41–49. 10.1016/s0006-8993(01)02023-6 11259758

[B27] Herring B. E. Nicoll R. A. (2016). Long-term potentiation: from CaMKII to AMPA receptor trafficking. Annu. Rev. Physiol. 78, 351–365. 10.1146/annurev-physiol-021014-071753 26863325

[B28] Honda Y. Ishizuka N. (2015). Topographic distribution of cortical projection cells in the rat subiculum. Neurosci. Res. 92, 1–20. 10.1016/j.neures.2014.11.011 25514386

[B29] Inostroza M. Cid E. Brotons-Mas J. Gal B. Aivar P. Uzcategui Y. G. (2011). Hippocampal-dependent spatial memory in the water maze is preserved in an experimental model of temporal lobe epilepsy in rats. PLoS One 6:e22372. 10.1371/journal.pone.0022372 21829459 PMC3144225

[B30] Ishizuka N. (2001). Laminar organization of the pyramidal cell layer of the subiculum in the rat. J. Comp. Neurol. 435, 89–110. 10.1002/cne.1195 11370013

[B31] Kandel E. R. (2001). The molecular biology of memory storage: a dialogue between genes and synapses. Science 294, 1030–1038. 10.1126/science.1067020 11691980

[B32] Knopp A. Frahm C. Fidzinski P. Witte O. W. Behr J. (2008). Loss of GABAergic neurons in the subiculum and its functional implications in temporal lobe epilepsy. Brain 131, 1516–1527. 10.1093/brain/awn095 18504292

[B33] Knopp A. Kivi A. Wozny C. Heinemann U. Behr J. (2005). Cellular and network properties of the subiculum in the pilocarpine model of temporal lobe epilepsy. J. Comp. Neurol. 483, 476–488. 10.1002/cne.20460 15700275

[B34] Laurent-Demir C. Decorte L. Jaffard R. Mons N. (2000). Differential regulation of Ca^2+^-calmodulin stimulated and Ca^2+^-insensitive adenylyl cyclase messenger RNA in intact and denervated mouse hippocampus. Neuroscience 96, 267–274. 10.1016/s0306-4522(99)00554-0 10683567

[B35] Lisman J. Yasuda R. Raghavachari S. (2012). Mechanisms of CaMKII action in long-term potentiation. Nat. Rev. Neurosci. 13, 169–182. 10.1038/nrn3192 22334212 PMC4050655

[B36] Malenka R. C. Bear M. F. (2004). LTP and LTD: an embarrassment of riches. Neuron 44, 5–21. 10.1016/j.neuron.2004.09.012 15450156

[B37] Marchi N. Oby E. Batra A. Uva L. De Curtis M. Hernandez N. (2007). *in vivo* and *in vitro* effects of pilocarpine: relevance to ictogenesis. Epilepsia 48, 1934–1946. 10.1111/j.1528-1167.2007.01185.x 17645533 PMC3900294

[B38] Martin S. J. Grimwood P. D. Morris R. G. M. (2000). Synaptic plasticity and memory: an evaluation of the hypothesis. Annu. Rev. Neurosci. 23, 649–711. 10.1146/annurev.neuro.23.1.649 10845078

[B39] Mattia D. Hwa G. G. C. Avoli M. (1993). Membrane properties of rat subicular neurons *in vitro*. J. Neurophysiol. 70, 1244–1248. 10.1152/jn.1993.70.3.1244 8229171

[B40] McClelland J. L. McNaughton B. L. O’Reilly R. C. (1995). Why there are complementary learning systems in the hippocampus and neocortex: insights from the successes and failures of connectionist models of learning and memory. Psychol. Rev. 102, 419–457. 10.1037/0033-295x.102.3.419 7624455

[B41] Menendez de la Prida L. Suarez F. Pozo M. A. (2003). Electrophysiological and morphological diversity of neurons from the rat subicular complex *in vitro*. Hippocampus 13, 728–744. 10.1002/hipo.10123 12962317

[B42] Menendez de la Prida L. Totterdell S. Gigg J. Miles R. (2006). The subiculum comes of age. Hippocampus 16, 916–923. 10.1002/hipo.20220 17016818

[B43] Miles B. Y. R. Wong R. K. S. (1987). Inhibitory control of local exitatory circuits in the guinea-pig hippocampus. J. Physiol. 388, 611–629. 10.1113/jphysiol.1987.sp016634 3656200 PMC1192568

[B44] Mulkey R. M. Malenka R. C. (1992). Mechanisms underlying induction of homosynaptic long-term depression in area CA1 of the hippocampus. Neuron 9, 967–975. 10.1016/0896-6273(92)90248-c 1419003

[B45] Naber P. A. Witter M. P. (1998). Subicular efferents are organized mostly as parallel projections: a double-labeling, retrograde-tracing study in the rat. J. Comp. Neurol. 393, 284–297. 10.1002/(sici)1096-9861(19980413)393:3<284::aid-cne2>3.0.co;2-y 9548550

[B46] Naber P. A. Lopes F. H. Witter M. P. (2001). Reciprocal connections between the entorhinal cortex and hippocampal fields CA1 and the subiculum are in register with the projections from CA1 to the subiculum. Hippocampus 11, 99–104. 10.1002/hipo.1028 11345131

[B47] Naber P. A. Witter M. P. Lopes Da Silva F. H. (2006). Networks of the hippocampal memory system of the rat: the pivotal role of the subiculum. Annu. N Y Acad. Sci. 911, 392–403. 10.1111/j.1749-6632.2000.tb06739.x 10911887

[B48] Nicholls J. G. Purves D. (1970). Monosynaptic chemical and electrical connexions between sensory and motor cells in the central nervous system of the leech. J. Physiol. 209, 647–667. 10.1113/jphysiol.1970.sp009184 5499801 PMC1395546

[B49] Nicoll R. A. (2017). A brief history of long-term potentiation. Neuron 93, 281–290. 10.1016/j.neuron.2016.12.015 28103477

[B50] O’Mara S. (2006). Controlling hippocampal output: the central role of subiculum in hippocampal information processing. Behav. Brain Res. 174, 304–312. 10.1016/j.bbr.2006.08.018 17034873

[B51] O’Mara S. M. Commins S. Anderson M. Gigg J. (2001). The subiculum: a review of form, physiology and function. Prog. Neurobiol. 64, 129–155. 10.1016/s0301-0082(00)00054-x 11240210

[B52] Sanabria E. R. G. Su H. Yaari Y. (2001). Initiation of network bursts by Ca2+-dependent intrinsic bursting in the rat pilocarpine model of temporal lobe epilepsy. J. Physiol. 532, 205–216. 10.1111/j.1469-7793.2001.0205g.x 11283235 PMC2278527

[B53] Squire L. R. Stark C. E. L. Clark R. E. (2004). The medial temporal lobe. Annu. Rev. Neurosci. 27, 279–306. 10.1146/annurev.neuro.27.070203.144130 15217334

[B54] Staff N. P. Jung H. Y. Thiagarajan T. Yao M. Spruston N. (2000). Resting and active properties of pyramidal neurons in subiculum and CA1 of rat hippocampus. J. Neurophysiol. 84, 2398–2408. 10.1152/jn.2000.84.5.2398 11067982

[B55] Staley K. J. Otis T. S. Mody I. (1992). Membrane properties of dentate gyrus granule cells: comparison of sharp microelectrode and whole-cell recordings. J. Neurophysiol. 67, 1346–1358. 10.1152/jn.1992.67.5.1346 1597717

[B56] Stewart M. Wong K. S. (1993). Intrinsic properties and evoked responses of guinea pig subicular neurons *in vitro*. J. Neurophysiol. 70, 232–245. 10.1152/jn.1993.70.1.232 8395577

[B57] Tamamaki N. Nojyo Y. (1990). Disposition of the slab-like modules formed by axon branches originating from single CA1 pyramidal neurons in the rat hippocampus. J. Comp. Neurol. 291, 509–519. 10.1002/cne.902910403 2329188

[B58] Taube J. (1993). Electrophysiological properties of neurons in the rat subiculum *in vitro*. Exp. Brain Res. 96, 304–318. 10.1007/bf00227110 7903643

[B59] Uva L. Librizzi L. Marchi N. Noe F. Bongiovanni R. Vezzani A. (2008). Acute induction of epileptiform discharges by pilocarpine in the *in vitro* isolated guinea-pig brain requires enhancement of blood-brain barrier permeability. Neuroscience 151, 303–312. 10.1016/j.neuroscience.2007.10.037 18082973 PMC2774816

[B60] Villacres E. C. Wong S. T. Chavkin C. Storm D. R. (1998). Type I adenylyl cyclase mutant mice have impaired mossy fiber long-term potentiation. J. Neurosci. 18, 3186–3194. 10.1523/jneurosci.18-09-03186.1998 9547227 PMC6792654

[B61] Wang H. Storm D. R. (2003). Calmodulin-regulated adenylyl cyclases: cross-talk and plasticity in the central nervous system. Mol. Pharm. 63, 463–468. 10.1124/mol.63.3.463 12606751

[B62] Wang H. Chan G. C. Athos J. Storm D. R. (2002). Synaptic concentration of type-I adenylyl cyclase in cerebellar neurons. J. Neurochem. 83, 946–954. 10.1046/j.1471-4159.2002.01206.x 12421367

[B63] Wang H. Gong B. Vadakkan K. I. Toyoda H. Kaang B. K. Zhuo M. (2007). Genetic evidence for adenylyl cyclase 1 as a target for preventing neuronal excitotoxicity mediated by N-methyl-D-aspartate receptors. J. Biol. Chem. 282, 1507–1517. 10.1074/jbc.m607291200 17121841

[B64] Wellmer J. Su H. Beck H. Yaari Y. (2002). Long-lasting modification of intrinsic discharge properties in subicular neurons following status epilepticus. Eur. J. Neurosci. 16, 259–266. 10.1046/j.1460-9568.2002.02086.x 12169108

[B65] Witter M. P. (2006). Connections of the subiculum of the rat: topography in relation to columnar and laminar organization. Behav. Brain Res. 174, 251–264. 10.1016/j.bbr.2006.06.022 16876886

[B66] Wong S. T. Athos J. Figueroa X. A. Pineda V. V. Schaefer M. L. Chavkin C. C. (1999). Calcium-stimulated adenylyl cyclase activity is critical for hippocampus-dependent long-term memory and late phase LTP. Neuron 23, 787–798. 10.1016/s0896-6273(01)80036-2 10482244

[B67] Wozny C. Kivi A. Lehmann T.-N. Dehnicke C. Heinemann U. Behr J. (2003). Comment on “On the origin of interictal activity in human temporal lobe epilepsy *in vitro*. Science 301:463. 10.1126/science.1084237 12881553

[B68] Wozny C. Maier N. Fidzinski P. Breustedt J. Behr J. Schmitz D. (2008a). Differential cAMP signaling at hippocampal output synapses. J. Neurosci. 28, 14358–14362. 10.1523/jneurosci.4973-08.2008 19118168 PMC6671250

[B69] Wozny C. Maier N. Schmitz D. Behr J. (2008b). Two different forms of long-term potentiation at CA1-subiculum synapses. J. Physiol. 586, 2725–2734. 10.1113/jphysiol.2007.149203 18403426 PMC2536578

[B70] Wu Z. L. Thomas S. A. Villacres E. C. Xia Z. Simmons M. L. Chavkin C. (1995). Altered behavior and long-term potentiation in type I adenylyl cyclase mutant mice. Proc. Nat. Acad. Sci. U S A. 92, 220–224. 10.1073/pnas.92.1.220 7816821 PMC42849

[B71] Yi F. DeCan E. Stoll K. Marceau E. Deisseroth K. Lawrence J. J. (2015). Muscarinic excitation of parvalbumin-positive interneurons contributes to the severity of pilocarpine-induced seizures. Epilepsia 56, 297–309. 10.1111/epi.12883 25495999 PMC4339470

[B72] Zeineh M. M. Engel S. A. Thompson P. M. Bookheimer S. Y. (2003). Dynamics of the hippocampus during encoding and retrieval of face-name pairs. Science 299, 577–580. 10.1126/science.1077775 12543980

[B73] Zucker R. S. Regehr W. G. (2002). Short-term synaptic plasticity. Annu. Rev. Physiol. 64, 355–405. 10.1146/annurev.physiol.64.092501.114547 11826273

